# Editorial: The epidemiologic triads in aquaculture: host, pathogen and environment

**DOI:** 10.3389/fimmu.2023.1305784

**Published:** 2023-11-09

**Authors:** Chou Min Chong, Po-Tsang Lee, Krzysztof Rakus, Eakapol Wangkahart

**Affiliations:** ^1^ Laboratory of Sustainable Aquaculture (AquaLab), International Institute of Aquaculture and Aquatic Sciences (I-AQUAS), Universiti Putra Malaysia, Port Dickson, Negeri Sembilan, Malaysia; ^2^ Department of Aquaculture, National Taiwan Ocean University, Keelung City, Taiwan; ^3^ Department of Evolutionary Immunology, Institute of Zoology and Biomedical Research, Faculty of Biology, Jagiellonian University, Krakow, Poland; ^4^ Laboratory of Fish Immunology and Nutrigenomics, Applied Animal and Aquatic Sciences Research Unit, Division of Fisheries, Faculty of Technology, Mahasarakham University, Kantarawichai, Maha Sarakham, Thailand

**Keywords:** host, pathogen, environment, dynamic interplay, fish immunity

The intricate interplay among hosts, pathogens, and environmental variables is pivotal in the realm of aquatic health, orchestrating the physiological and immunological responses of aquatic species and directing the outcomes of infectious diseases. This multifaceted relationship is characterized by perpetual adaptations of both hosts and pathogens, each striving to outmaneuver the other, with environmental conditions adding an additional layer of complexity. The host, in its pursuit to maintain physiological equilibrium, optimizes immune responses to fend off pathogens effectively while avoiding damage induced by the overexpression of inflammatory genes. In contrast, pathogens engineer sophisticated strategies to infiltrate and bypass host defenses, manipulating host immune responses to establish infection.

In this dynamic ecosystem, hosts evolve to counter pathogens, modulating responses to maintain equilibrium and avoid overreaction. Achieving this equilibrium is essential to counter pathogens without overwhelming the host or inducing harm. This delicate balance is exemplified by studies conducted by Xie et al. and Zhao et al., focusing on golden pompano (*Trachinotus ovatus*) and zebrafish (*Danio rerio*), respectively. Xie et al. explored the expression of the Double Ig Interleukin-1 Receptor-Related Molecule (DIGIRR) in golden pompano, which shares homology with the mammalian Single Immunoglobulin (Ig) Interleukin-1 Receptor-Related Molecule (SIGIRR). DIGIRR has relatively high expression in the intestine, liver, head kidney (HK), and spleen, and relatively low expression in the heart and muscle, indicating its foundational role in maintaining physiological stability. However, the introduction of pathogens induces specific alterations in DIGIRR expressions, tailored to the nature of the encountered pathogen. These alterations are crucial in preventing excessive inflammation, highlighting the adaptability of the immune system and the pivotal role of DIGIRR in modulating inflammatory responses and maintaining physiological equilibrium in the host.

Zhao et al. elucidated the role of PLAAT1, a phospholipase A1/2 belonging to the phospholipase A/acyltransferase (PLAAT) family, revealing how pathogens, such as viruses, utilize strategies to evade host immune responses. Maintaining a balanced IFN response is essential during and after viral clearance to avoid excessive damage to the host. Zhao et al. disclosed that PLAAT1 interacts with IRF3 and IRF7, initiating their degradation and mitigating IFN expression. This negative regulation of IFN response is crucial for optimizing the host’s immune response and restoring homeostasis. Host-pathogen interactions are not unidirectional, as revealed by Zhang et al., who illuminated the strategies employed by pathogens to actively counter host immune responses, highlighting the bidirectional adaptability inherent in these interactions. Their study focused on Edwardsiella piscicida-induced enteritis in the big-belly seahorse (*Hippocampus abdominalis*) and identified 15 core virulence factors that collaborated to orchestrate the remodeling of intestinal microorganisms and host metabolism, ultimately leading to enteritis. These virulence factors played crucial roles in facilitating the pathogen's evasion of host defenses by enhancing its motility, adherence, and intracellular survival, while also aiding in the evasion of phagocytosis. This research emphasizes the dynamic and evolving nature of host-pathogen interactions, where both sides continually adapt to maintain equilibrium.

Environmental factors significantly influence the immune profiles of aquatic species, shaping their responses to diverse challenges. Studies conducted by Kato et al., Pan et al., and Sun et al. provide valuable insights into this phenomenon. Kato et al. examined the impact of water temperature, specifically at 22°C compared to 12°C, on ayu (*Plecoglossus altivelis*), revealing a substantial effect on the expression of proinflammatory genes, including IL-1β and IL-8. Elevated temperatures were found to upregulate these genes, influencing the fish’s susceptibility to disease. Similarly, Pan et al. investigated the effects of varying environmental pH levels, focusing on the acidity of the water (pH 5, 6, and 7), on *Oreochromis niloticus*. Remarkably, changes in pH induced the expression of pivotal proinflammatory genes such as TNF-α, IL-1β, IL-8, and IL-12 over a two-week period. Sun et al. further elucidated the role of temperature in the immune response and disease susceptibility of flounder (*Paralichthys olivaceus*) during *Edwardsiella tarda* infection, revealing that higher temperatures exacerbate the bacterial load in the spleen, thereby influencing the ability of flounder to combat bacterial infection. These findings collectively underscore the adaptability of aquatic species in responding to environmental cues and alterations, revealing a consistent trend in the upregulation of proinflammatory genes across different environmental stressors.

The epidemiologic triads, consisting of host, pathogen, and environment, exert a profound influence on health measures, as demonstrated by the studies of Kato et al. and Sun et al. Kato et al. observed that the relative percent survival of vaccinated fish reared at higher temperatures (15°C and 22°C) was lower than those reared at 12°C when challenged with *Flavobacterium psychrophilum*, highlighting the impact of temperature on vaccination effectiveness. Sun et al. provided additional insights into the role of temperature in *E. tarda* infection in flounder, demonstrating that temperature significantly influences the ability of flounder to block bacterial infection, with a comparative metabolomic analysis revealing distinct metabolite responses to infection under different temperature conditions. They identified specific Metabolite Markers of Temperature Effect (MMTEs), which are significantly differential metabolites (SDMs) reflecting the metabolic alterations due to infection and temperature, categorized into various metabolic pathways like Aminoacyl-tRNA biosynthesis and Phenylalanine metabolism. The supplementation of selected MMTEs significantly modulates pro-inflammatory gene expression and influences infection dynamics, thereby improving the survival of flounder during *E. tarda* infection. This underscores the importance of a comprehensive approach in aquatic health management, incorporating all elements of the epidemiologic triads, to enhance the resilience of aquatic species in the face of evolving health challenges, and it emphasizes the potential of metabolic interventions in managing disease and improving survival rates in aquatic species.

The dynamic interrelations between hosts, pathogens, and the environment form a complex nexus that significantly impacts the health and resilience of aquatic species. A comprehensive approach is necessitated in aquatic health management, incorporating all elements of the epidemiologic triads, to enhance the resilience of aquatic species in the face of evolving health challenges. Future research and interventions in this field should continue to explore the multifaceted interactions between host, pathogen, and environment, aiming to develop tailored strategies and solutions to address the unique needs and challenges of different aquatic species and ecosystems.

## Author contributions

CC: Writing – original draft, Writing – review & editing. PL: Writing – original draft, Writing – review & editing. KR: Writing – original draft, Writing – review & editing. EW: Writing – original draft, Writing – review & editing.

